# Socioeconomic Differences in Exposure to Tobacco Smoke Pollution (TSP) in Bangladeshi Households with Children: Findings from the International Tobacco Control (ITC) Bangladesh Survey

**DOI:** 10.3390/ijerph8030842

**Published:** 2011-03-15

**Authors:** Abu S. Abdullah, Sara C. Hitchman, Pete Driezen, Nigar Nargis, Anne C.K. Quah, Geoffrey T. Fong

**Affiliations:** 1 School of Public Health, Guangxi Medical University, 22 Shuangyong Road, Nanning 530021, Guangxi, China; 2 Department of Medicine (MISU), Boston University School of Medicine, 801 Massachusetts Avenue (2nd floor), Boston, MA 02118, USA; 3 Department of Psychology, University of Waterloo, 200 University Avenue West, Waterloo, Ontario N2L3G1, Canada; E-Mails: schitchm@uwaterloo.ca (S.C.H.); ackquah@uwaterloo.ca (A.C.K.Q.); gfong@uwaterloo.ca (G.T.F.); 4 Propel Centre for Population Health Impact, University of Waterloo, 200 University Avenue West, Waterloo, Ontario N2L3G1, Canada; E-Mail: prdriezen@uwaterloo.ca; 5 Department of Economics, University of Dhaka, Arts Building, Room 4057, Dhaka-1000, Bangladesh; E-Mail: nigar_nargis@econdu.ac.bd; 6 Ontario Institute for Cancer Research, MaRS Centre, South Tower, 101 College Street, Suite 800, Toronto, Ontario M5G0A3, Canada

**Keywords:** tobacco smoke pollution (TSP), second hand smoke (SHS), smoking restrictions, children, Bangladesh

## Abstract

This study assessed the pattern of exposure to tobacco smoke pollution (TSP; also known as, secondhand smoke) in Bangladeshi households with children and examined the variations in household smoking restrictions and perception of risk for children’s exposure to TSP by socioeconomic status. We interviewed 1,947 respondents from Bangladeshi households with children from the first wave (2009) of the International Tobacco Control (ITC) Bangladesh Survey. 43.5% of the respondents had complete smoking restrictions at home and 39.7% were very or extremely concerned about TSP risk to children’s health. Participants with lower level of education were significantly less likely to be concerned about the risk of TSP exposure to children’s health and less likely to adopt complete smoking restrictions at home. Logistic regression revealed that the predictors of concern for TSP exposure risk were educational attainment of 1 to 8 years (OR = 1.94) or 9 years or more (OR = 4.07) and being a smoker (OR = 0.24). The predictors of having complete household smoking restrictions were: urban residence (OR = 1.64), attaining education of 9 years or more (OR = 1.94), being a smoker (OR = 0.40) and being concerned about TSP exposure risk to children (OR = 3.25). The findings show that a high proportion of adults with children at home smoke tobacco at home and their perceptions of risk about TSP exposure to children’s health were low. These behaviours were more prevalent among rural smokers who were illiterate. There is a need for targeted intervention, customized for low educated public, on TSP risk to children’s health and tobacco control policy with specific focus on smoke-free home.

## 1. Introduction

Exposure to tobacco smoke pollution (TSP), also known as “second-hand smoke (SHS)” exposure or “passive smoking” is increasingly being recognized as a major public health threat. Worldwide, the World Health Organization (WHO) estimated that 40% of children were exposed to TSP in 2004. The estimated attributable deaths due to TSP totaled 603,000, of which 28% were estimated to be children. Children accounted for 61% of DALYS (Disability Adjusted Life Years) lost worldwide; with the largest disease burden due to lower respiratory tract infections in children under 5 years of age [1]. Chronic exposure to TSP in children is associated with an increased risk of a range of adverse outcomes, including lower respiratory tract infections, wheezing, coughing, middle ear infections and sudden infant death syndrome [2–4]. Furthermore, childhood TSP exposure decreases adult lung function even in individuals who never smoked themselves [5]. These adverse effects of TSP have led to policies, in many countries, prohibiting smoking in a range of public settings including workplaces [6], and recreational facilities [7]. Knowledge of and attitudes towards TSP was associated with supporting smoking restrictions in a number of studies [8–10]. Awareness of the health risks of TSP was positively associated with support for smoke-free public places among the Chinese adults [8,10]. Chen *et al.*, found that awareness of the health risks of TSP was positively associated with support for smoke-free public places among Taiwanese adults [9]. Also, higher education was significantly associated with the support for smoke-free public places in these studies [8,10,11].

With the widespread establishment of smoke-free workplaces and public venues, the home is becoming the predominant source of exposure to TSP among children and non-smoking adults. [1,2,12,13]. Hence, interest has increased in studying the pattern and practices of household exposure to TSP [14–16]. However, the vast majority of available information concerning household exposure to TSP and measures to reduce exposure comes from studies conducted mostly in developed or high income countries, and data from developing or low income countries is limited. Understanding the impact of knowledge and attitudes towards TSP exposure and how this impact might vary as a function of socioeconomic status (SES) would be useful to guide targeted policy development in low and middle-income countries (LMICs).

The general objective of the present study was to examine the prevalence of TSP exposure as well as knowledge and attitudes toward TSP exposure in Bangladesh. With a population of 144.5 million, Bangladesh is one of the world’s most densely populated countries, with over 22 million adult smokers [17]. The high prevalence of smoked tobacco use among adults (23.0%; male: 44.7%, female: 1.5%) in Bangladesh [17], means that a large number of children are exposed to TSP at home and/or in other public venues. Additionally, because there are SES variations in smoking behavior in Bangladesh [17], it may be that children from lower SES groups are exposed to TSP more frequently than children from high SES groups due to variations in household smoking restrictions [18].

The aim of this study was to assess the pattern of exposure to TSP in Bangladeshi households and examine the variations in household smoking restrictions and perception of risk for children’s exposure to TSP by SES.

## 2. Methods

### 2.1. Setting

The International Tobacco Control (ITC) Bangladesh Survey is a prospective cohort survey of a nationally representative sample of smokers and non-smokers conducted in all six administrative divisions of Bangladesh: Barisal, Chittagong, Dhaka, Khulna, Rajshahi, and Sylhet. The target population of the ITC Bangladesh Survey consists of users and non-users of tobacco who are 15 years or older. The ITC Bangladesh Survey, as with all ITC Surveys being conducted in 20 countries, was designed to evaluate the psychosocial and behavioural effects of tobacco control policies in Bangladesh as well as to understand factors that are related to the natural history of tobacco use over time. [19] The ITC Bangladesh Survey was designed as a follow-up study of the 2004–05 WHO Study, “Impact of Tobacco-related Illnesses in Bangladesh”, which was conducted soon after Bangladesh’s ratification of the Framework Convention on Tobacco Control (FCTC) but before any policy action had taken place. The ITC Bangladesh Wave 1 Survey data were collected between February and May 2009. Survey data collected between February and May 2009 was a contribution to the ongoing surveillance efforts among adults and youth in assessing the impact of the Tobacco Control Act, which was enacted in 2005 and whose provisions were implemented in 2006, including, enhanced warning labels, smoke-free legislation, and advertising and promotion restrictions.

### 2.2. Sampling

The ITC Bangladesh Wave 1 Survey is a nationally representative probability sample of tobacco users and non-users of tobacco selected through a multi-stage clustered sampling design (sampling with probability proportional to population size at the levels of district, upazila/thana, village/ward). A total of 94,485 adults age 15 and older from 31,689 households were enumerated to establish an accurate sampling frame from which survey participants would be drawn. For the national sample, 23 districts out of the 64 districts covering Bangladesh were selected, 20 of them using probability proportional to population size. Two districts were selected purposively to include tribal populations (Garo and Chakma) and one district was selected to cover one land port that is used for cross-border trade of tobacco products. A total of 40 upazilas from the 23 districts, and two villages from each upazila were selected, again with probability proportional to size. A total of 40 upazilas from the 23 districts, and two villages from each upazila were selected, again with probability proportional to size. Thus, a total of 80 villages/wards were selected for the national sample. In addition, six urban slum areas within the city of Dhaka and its surrounding areas were selected to conduct the survey among the floating and urban poor population (*i.e.*, slum sample).

A total of 25 households per village were selected based on the SES and smoking status of household members. Thus at the end of the census, 2,000 households had been selected from 80 villages for the cohort survey. Household members aged 15 years and older were sampled from within a household to participate in the survey. From households with smokers, all available smokers, and one non-smoker was randomly selected for interview. From households without smokers, we randomly selected one non-smoker. Thus the total number of non-smoker respondents was fixed at 25, one from each sample household. The total number of smoker respondents varied from village to village depending on the smoking prevalence of that area and the availability of respondents for interview. For the slum sample, the interviewers started randomly at one end of each slum area and continued interviewing each household in a row until they met the target of the designated number of households from that area. The households were enumerated and surveyed at the same visit. The interviewers selected one non-smoker randomly and all smokers from each household. The stratification of households based on housing condition was not followed for the slum sample.

#### Sampling Weights

For each household enumerated in the census, we have constructed a village-level household weight which was used to construct a national level household weight. Then, for each household where an interview was conducted, we constructed a national level household weight, consistent with the weights for enumerated households. For each individual, an individual weight was then computed within his/her household. The product of interview household weight and individual within-household weight was calibrated to sum to assumed population numbers in groups defined by a combination of geography and demographics.

### 2.3. Data Collection and Management

A standardized Bengali questionnaire was used for data collection. The survey was also administered in Garo and Chakma for the tribal population. A total of 5,763 (3,107 smokers and 2,656 non-smokers) face-to-face interviews were conducted. Of these 5,763 subjects, 1,947 adults who reported having a child (13 years or younger) living in the household were included in the analyses for this paper.

Data entry was done in parallel with the field-work. In order to control the quality of the data collection process a multistage monitoring system was used including unannounced field visits to monitor interviews by the project manager and field coordinator, calling randomly picked households to verify the information that interviewers filled in the survey form, and cross-checking of all completed forms by field supervisors daily to ensure that they had been properly completed.

Two data analysts continuously ran routine checks on the data sets, informing the field coordinator and project manager about any problems that might be present in data reporting and collection. In consultation with the investigators, the project manager then decided on the best method(s) for correcting errors and for communicating to all the field staff using a hotline mobile phone network. As the fieldwork proceeded, the feedback gathered from the already entered data sets helped the field staff to learn from the past omissions and improve on the data collection process.

Written consent was obtained from those who can read and write; others gave verbal consent.

## 3. Measures

Details of the measures used in this study are briefly described below. These measures have been used in prior research studies in other international settings [10,12,20].

*Demographics.* Respondents’ demographic information was collected as part of the overall survey, including, gender, age, residence (rural, urban, slum), marital status, monthly household income, and education. Information about the number of children 13 years old or younger in the home, and the age of the youngest child was also collected. Household enumeration forms were completed to assess the number of adult smokers and non-smokers aged 15 years and older present in each household. See Table 1 for further details on variable categories used in the analyses.

*Smoking Behaviour (smokers only).* Respondents were asked about their smoking status, including, type of tobacco smoked (cigarette, bidi, or, dual user), sticks smoked per day, if they had ever attempted to quit smoking, and if they attempted to quit in the past year. Cigarette and bidi users all reported that they smoked at least weekly at the time of surveying.

*Tobacco Smoke Pollution Exposure (TSP)—Knowledge and Opinions on Restrictions.* Knowledge of the health consequences of TSP exposure was assessed, along with opinions towards smoking restrictions. To measure knowledge of the health consequences of TSP exposure, respondents were asked: “Based on what you know or believe, does second hand smoking cause…?” Respondents were then read a list of diseases. Measures from the list included in the present study were: lung cancer in non-smokers, and asthma in children. To measure opinions on smoking restrictions, respondents were asked: “For each of the following public places, please tell me if you think smoking should not be allowed in any indoor areas, should be allowed only in some indoor areas, or no rules or restrictions?” The list included: hospitals, workplaces, restaurants or tea stalls, public transportation vehicles, and schools/colleges/universities. See Table 2 for further details on variable categories used in the analyses.

*Other Smoking Related Measures.* Respondents were also asked, “Out of your five closest friends, how many of them are smokers?” (0 to 5). To measure knowledge of the addictive nature of tobacco, respondents were asked: “Please tell me whether you strongly agree, agree, neither agree nor disagree, disagree, or disagree strongly with the following statement. The statement read: Smoking is addictive.

*Dependent Variables*. Two key dependent variables were examined: (i) concern that smoking in the presence of children harms their health, and (ii), household smoking restrictions. Respondents’ concern that smoking in the presence of children harms children’s health was assessed by asking, “How concerned are you that smoking in the presence of your children will hurt your children’s health?” Response categories were: not concerned, a little concerned, moderately concerned, very concerned and extremely concerned, no children in my household, and I do not smoke in the presence of my children. Respondents, who said, ‘I do not smoke in the presence of my children,’ were assigned to the extremely concerned category. Respondents who said there were no children in their household were assigned missing values. Non-smokers were asked a slightly different version of the question, “How concerned are you that your children’s health will be hurt if people smoke in their presence?” Response categories were: not concerned, a little concerned, moderately concerned, very concerned and extremely concerned, I have no children, and people do not smoke in the presence of my children. Respondents, who said, ‘people do not smoke in the presence of my children,’ were assigned to the extremely concerned category. Respondents, who said, ‘I have no children,’ were assigned missing values.

To measure household restrictions on smoking, we asked: “Which of the following best describes smoking inside your home?” Response categories were: smoking is not allowed in any indoor room inside home (*i.e.*, complete restrictions), smoking is allowed only in some rooms inside home (*i.e.*, partial restrictions), and no rules or restrictions (*i.e.*, no restrictions). Dependent variables were dichotomized for logistic regression modelling. Concern that smoking in the presence of children will harm their health was dichotomized as “very/extremely” concerned *vs.* otherwise while household smoking restrictions was dichotomized as “complete restrictions” *vs.* otherwise.

## 4. Data Analyses

SAS 9.2 was used for the analyses. Characteristics of respondents are presented by smoking status (unweighted). The two dependent measures were: (i) concern that smoking in the presence of children harms their health, and (ii) home smoking restrictions. Independent variables were: (i) demographics, (ii) smoking behaviour, (iii) knowledge and opinions of TSP, and (vi) other smoking related measures. Education was used as a proxy for SES, because tests of multi-collinearity (data not shown) showed that education was a better fit in the models than monthly household income.

The study consisted of four main sets of analyses: (1) chi-square tests were used to examine associations between smoking status, and knowledge and opinions regarding TSP, the addictiveness of smoking, and the two dependent measures. (2) Chi-square tests were used to examine associations between level of education (SES), and all independent and dependent variables. (3) Chi-square tests were used to examine the association between the two dependent variables. (4) Logistic regression was used to examine the predictors of the two dependent variables. All demographic variables (with the exception of income), and smoking status were included as predictors in the logistic regression models. Concern that smoking in the presence of children harms children’s health was included as an additional predictor variable in the regression analyses that examined predictors of home smoking restrictions.

Respondents with missing data or who gave refusals or don’t know responses were set to ‘missing values” and excluded from the analyses. The one exception was the health knowledge questions where don’t know responses were retained.

## 5. Results

### 5.1. Demographic Characteristics

Of the 5,763 people interviewed in Wave 1 of the ITC Bangladesh Survey, 1,947 (42%) reported having at least one child aged 13 or younger living in their homes. Of these, 27% (n = 532) were non-smokers while the remainder were smokers of cigarettes (51%), bidis (9%) or both cigarettes and bidis (13%). In general, the demographic characteristics of the participants differed by smoking status (see Table 1). Smokers were more likely than non-smokers to be male, aged 40 or older, married, to reside in urban slum areas, to be low income and illiterate (Table 1).

### 5.2. Knowledge of Harms Caused by TSP and Opinion on Smoke Free Policies

Thirty-eight percent (773/1,947) of the respondents were very or extremely concerned about the TSP risk to children’s health and 43.5% (847/1,947) of respondents had a complete ban on smoking at home. As shown in Table 2, support for smoke-free policies and knowledge about TSP also differed between smokers and non-smokers. While smokers and non-smokers alike supported complete smoking bans in hospitals and public transport, a smaller percentage of smokers supported complete smoking bans in workplaces (84% of smokers *vs.* 92% of non-smokers) and restaurants (69% of smokers *vs.* 87% of non-smokers). A somewhat smaller percentage of smokers than non-smokers were aware that TSP causes lung cancer in non-smokers (87% *vs.* 92%) and asthma in children (89.6% *vs.* 94%). Finally, a significantly smaller percentage of smokers were very or extremely concerned that smoking in the presence of their children could harm their health compared to non-smokers (31% *vs.* 66%, respectively, p < 0.001). Fewer smokers also had complete bans on smoking in their home compared to non-smokers (35% *vs.* 62%, respectively, p < 0.001).

Table 3 shows that support for smoke-free policies and knowledge about TSP also differed by educational category. In general, a smaller percentage of illiterate Bangladeshis supported complete smoking bans in workplaces (82%) and restaurants (69%) than the most educated Bangladeshis (90% and 78%, respectively). Fewer illiterate Bangladeshis were aware of the harmful effects of TSP as well: 83% of illiterate Bangladeshis knew that TSP causes lung-cancer in non-smokers compared to 95% of highly educated Bangladeshis while 85% of illiterate Bangladeshis knew that TSP causes asthma in children compared to 96% of highly educated Bangladeshis.

### 5.3. Household Smoking Conditions

Table 4 shows that education was strongly associated with concern for the effects of TSP exposure on children’s health and home smoking bans. Only 28% of illiterate Bangladeshis were very or extremely concerned that smoking in the presence of their children would harm their health compared to 45% of moderately educated and 66% of highly educated Bangladeshis (p < 0.001). Similarly, 37% of illiterate Bangladeshis had a complete smoking ban in their homes compared to 44% of moderately educated and 64% of highly educated Bangladeshis (p < 0.001). A higher proportion of illiterate Bangladeshis had two or more smokers in the same household compared to those who were highly educated (34.7% *vs.* 25.5%).

Table 5 shows that a high perception of risk (*i.e.*, very or extremely concerned) for children’s exposure to TSP was strongly associated with having a complete smoking ban at home (p < 0.001).

### 5.4. Predictors of the Perception of Risk for Children’s Exposure to TSP

Table 6 shows the results of logistic regression analysis about concern for children’s health. After controlling for other covariates, education was found to be a significant predictor of concern, and smoking status.

Compared to illiterate Bangladeshis, those having 1 to 8 years of education had 1.9 times greater odds of being very/extremely concerned that smoking in the presence of children harms their health (p < 0.001). Highly educated Bangladeshis had 4.1 times greater odds of being very concerned (p < 0.001). On the other hand, smokers had significantly lower odds of being very concerned that smoking in the presence of children harms their health compared to non-smokers (OR = 0.24, p < 0.001).

### 5.5. Predictors of Household Smoking Restrictions

Logistic regression analysis identified four predictors of complete household smoking bans: residence location, education, tobacco use and concern about smoking in the presence of children (Table 7).

Urban residents had significantly greater odds of having complete bans on smoking in their homes compared to rural residents (OR = 1.64, p = 0.05). Highly educated Bangladeshis also had significantly greater odds of having complete bans. Specifically, those with 9 or more years of education had 1.9 times greater odds of having a complete restriction compared to illiterate persons. Smokers had significantly lower odds of having complete bans than non-smokers (OR = 0.40, p < 0.001). Concern for children’s health was a strong predictor of having complete smoking bans in the home compared to those who were not concerned (OR = 3.25, p = 0.001).

## 6. Discussion

In a nationally representative sample of the Bangladeshi adults with children living in the household, this study examined the sociodemographic factors and TSP knowledge associated with household smoking restrictions and the perceptions of risk about children’s exposure to TSP. Our findings revealed that the prevalence of household TSP exposure to children was high (67%), and low education was associated with not having a complete smoking ban at home and having low concerns about the health risks of TSP exposure to children.

Data on SHS or TSP exposure among children in developing countries is scarce. However, some information is available from the Global Youth Tobacco Survey (GYTS) [21]. In the GYTS, data on the proportion of children reporting that they live in homes where others smoke was available for a number of developing countries, including China (56.1%), Indonesia (66.8%), and India (48.2%). Our finding of 67 % is comparable to some of these findings; however, much higher than the rate reported in the Bangladesh GYTS (34.7%) [21]. It should be noted that the GYTS data was based on children’s (aged 13–15 years) self-reported exposure, while we assessed exposure among children (aged 13 and under) from the adult’s self-report. It is obvious that younger children (*i.e.*, those 5 years or younger) stay more at home and regularly exposed to TSP. In a Hong Kong study among parents of young children (aged 4–5 years), 62.2% of parents had no home smoking restrictions [12]. However, the TSP exposure rate of 67% found in the present study was higher than the reported exposure rate among children at home in Canada (57%) [22] and Australia (50.5%) [23]. While methodological differences in how the data were collected across studies could be a contributing factor for this difference, it might also be due to the low overall smoking rate and increased awareness of the TSP risk among the public in these countries. Clean indoor air policies in these countries might also be a contributing factor [2].

Our findings showed that respondents possessed good knowledge about the harms of TSP with 87% knowing that TSP causes lung cancer in non-smokers and 89% knowing that TSP causes asthma in children. These findings are comparable to the findings of the Global Adult Tobacco Survey (GATS) conducted in Bangladesh in 2009. The 2009 GATS found that 93.4% adults knew that exposure to other people’s smoke causes serious illness in non-smokers [17]. Consistent with the findings of the GATS, we also found an increased level of knowledge among respondents with higher education. To maintain this high level of knowledge among the Bangladeshi public, existing tobacco control promotional campaigns should be continued on a regular basis. Graphic warning labels could be successful in reaching illiterate populations [24]. Because there are still differences in knowledge between smokers and non-smokers and by educational level, targeted campaigns, with customized messages, could be designed to reach illiterate populations. Positive attitudes towards smoking bans in hospitals, workplaces, public transport and schools (approximately 90% in favour of complete ban; with variations between smokers and non-smokers) suggests a strong basis for implementing smoke-free policies in Bangladesh.

In this study, low education and being a smoker were predictors of having lower concerns about the health effects of TSP exposure on children. It may be that illiterate smokers and non-smokers do not fully understand the health risks of TSP [25]. It is also possible that these smokers knowingly ignored the health risks of TSP and thought that TSP would not have any negative impact on children’s health. Given the fact that there is a significant relationship between perceptions of TSP risk and adopting home smoking restrictions, interventions should be designed to increase peoples’ perceptions of the risks about smoking and TSP exposure risk to promote smoke-free homes.

In the current study a number of factors predicted the implementation of complete household smoking restrictions among Bangladeshi adults, including being a slum resident, attaining a higher level of education (9 or more years of education), being non-smokers and those who had high perception of risk about TSP exposure to children’s health. Studies in other countries also identified some of these predictors [12,26,27]. Higher level of home smoking restrictions among the slum (p = 0.018) and urban (p = 0.05) residents may be due to the fact that these population groups are more likely to be well educated who belong to more affluent SES. Also, compared to the rural residents, slum and urban population are more exposed to health education messages from electronic or other media campaign, which may make them more proactive towards health protective behaviours. Lower levels of home smoking restrictions among those who were smokers suggest the need for smoking cessation services as in some cases, due to the addictive nature of tobacco use; smokers find it difficult to implement smoke-free environments [28]. The finding that those with higher levels of education are more likely to adopt home smoking restrictions suggests that home smoking restrictions may often be adopted in the context of social class [25], reflecting the need to consider SES in the design of interventions. This study had several limitations. First, because of the cross-sectional design of the study only associations could be explored without any causal relationship. Second, data were self-reported and subject to recall and reporting bias. However, studies have shown that parental self-report of TSP exposure of children is moderately related to either environmental or biological measures of TSP and has sufficient validity [29]. Finally, data were collected by trained interviewers who followed written interviewer guidelines. However, any difference between their understanding and explanation of the questions asked could result in bias in information collected. Despite these limitations, our study provides important information about the household smoking restrictions in Bangladeshi families with young children.

In conclusion, many young children in Bangladesh live in a house with adult smokers and no home smoking bans, exposing them to high levels of TSP. The perceptions of risk about children’s exposure to TSP are also low among Bangladeshi adults. Findings from this study suggest that innovative programs are needed to enhance the implementation of home smoking bans in Bangladeshi families. This is particularly important for the families who have young children. We believe that with the wider implementation of home smoking bans more children and non-smokers will be protected from the health risks of TSP exposure. [30]. Public health practitioners should work to enhance home smoking restrictions by focusing on the predictors identified in this study and targeting those who are smokers, rural residents, illiterate, and those with low perception of TSP risk. Future studies, possibly qualitative in nature, would be useful to understand the processes that families might go through in adopting home smoking restrictions and to identify facilitating factors or barriers.

## Figures and Tables

**Table 1.** Characteristics of ITC Bangladesh respondents having at least one child in the home (unweighted) (N = 1,947).

Characteristic		Non-smokers		Smokers		Overall		Rao-Scott χ^2^ Test
		Freq.	%	Freq.	%	Freq.	%	p-value
Sex	Male	193	36.3	1,378	97.4	1,571	80.7	<0.001
	Female	339	63.7	37	2.6	376	19.3	

Age (grouped)	15–24	64	12.0	31	2.2	95	4.9	<0.001
	25–39	212	39.8	471	33.3	683	35.1	
	40–54	134	25.2	522	36.9	656	33.7	
	55+	122	22.9	391	27.6	513	26.3	

Residence	Urban (non-slum areas)	145	27.3	314	22.2	459	23.6	<0.001
	Slums	44	8.3	365	25.8	409	21.0	
	Rural	343	64.5	736	52.0	1,079	55.4	

Marital status	Otherwise	111	21.3	113	8.0	224	11.6	<0.001
	Married	411	78.7	1,299	92.0	1,710	88.4	

Monthly household income	<5,000 taka	73	13.7	304	21.5	377	19.4	<0.001
	5,000 to <10,000 taka	144	27.1	672	47.5	816	41.9	
	10,000 taka or more	121	22.7	294	20.8	415	21.3	
	Not reported	194	36.5	145	10.2	339	17.4	

Education	Illiterate	143	26.9	498	35.2	641	33.0	<0.001
	1 to 8 years	268	50.5	724	51.2	992	51.0	
	9 years or more	120	22.6	192	13.6	312	16.0	

Number of children in the home	1	209	39.3	562	39.7	771	39.6	0.981
	2	189	35.5	503	35.5	692	35.5	
	3 or more	134	25.2	350	24.7	484	24.9	

Age of youngest child	0 to 5	340	63.9	917	64.8	1257	64.6	0.698
	6 to 13	192	36.1	498	35.2	690	35.4	

Number of friends who smoke	0 or 1	37	13.1	36	2.7	73	4.5	<0.001
	2 to 5	245	86.9	1,296	97.3	1,541	95.5	

Living with other adult smokers	No (other) smokers in home	233	43.8	798	56.4	1,031	53.0	0.056
	At least 1 (other) smoker in home	299	56.2	617	43.6	916	47.0	

Mean (SD) among smoked per day		NA		12.2 (8.3)		NA		NA

**Table 2.** Support for smoking bans in different venues and knowledge and attitudes about tobacco smoke pollution (weighted estimates), by smoking status (N = 1,947).

Measure		Non-smokers			Smokers			Overall			Rao-Scott χ^2^ Test
		Freq.	%	(95% CI)	Freq.	%	(95% CI)	Freq.	%	(95% CI)	p-value
Restrictions on smoking in hospitals	Complete ban	508	98.0	(95.0–99.5)	1,387	98.7	(97.5–99.4)	1,895	98.5	(97.3–99.2)	0.499
	Otherwise	6	2.0	(0.5–5.0)	19	1.3	(0.6–2.5)	25	1.5	(0.8–2.7)	

Restrictions on smoking in workplaces	Complete ban	457	91.6	(86.3–94.9)	1,174	83.9	(78.0–88.4)	1,631	86.7	(82.4–90.0)	0.017
	Otherwise	51	8.4	(5.1–13.7)	216	16.1	(11.6–22.0)	267	13.3	(10.0–17.6)	

Restrictions on smoking in restaurants	Complete ban	427	86.7	(80.2–91.3)	994	69.4	(64.6–73.7)	1,421	75.6	(71.7–79.2)	<0.001
	Otherwise	78	13.3	(8.7–19.8)	397	30.6	(26.3–35.4)	475	24.4	(20.9–28.3)	

Restrictions on smoking in public transport	Complete ban	518	99.9	(99.1–100.0)	1,387	99.0	(98.0–99.6)	1,905	99.3	(98.7–99.7)	0.002
	Otherwise	2	0.1	(0.0–0.9)	15	1.0	(0.4–2.0)	17	0.7	(0.3–1.3)	

Restrictions on smoking in schools	Complete ban	492	93.7	(89.1–96.4)	1,342	96.4	(94.2–97.9)	1,834	95.4	(92.8–97.3)	0.026
	Otherwise	29	6.3	(3.6–10.9)	61	3.6	(2.1–5.8)	90	4.6	(2.7–7.2)	

Smoked tobacco is addictive	Agree/Strongly Agree	506	97.0	(93.9–98.8)	1,330	95.5	(93.9–96.8)	1,836	96.0	(94.5–97.3)	0.255
	Otherwise	17	3.0	(1.2–6.1)	59	4.5	(3.2–6.1)	76	4.0	(2.7–5.5)	

Second-hand smoke causes lung cancer in non-smokers	Yes	470	92.0	(88.5–94.5)	1,219	86.6	(80.2–91.2)	1,689	88.6	(84.4–91.8)	0.004
	No	22	2.1	(1.0–3.7)	81	7.2	(3.9–12.8)	103	5.3	(3.1–8.8)	
	Don’t know	39	6.0	(3.7–9.3)	82	6.2	(4.3–8.8)	121	6.1	(4.5–8.2)	

Second-hand smoke causes asthma in children	Yes	481	93.9	(90.9–96.0)	1,251	89.6	(84.2–93.3)	1,732	91.2	(87.2–94.0)	<0.001
	No	8	0.9	(0.3–2.2)	59	4.9	(2.3–9.0)	67	3.4	(1.7–6.1)	
	Don’t know	43	5.2	(3.3–8.0)	70	5.5	(3.3–9.0)	113	5.4	(3.4–8.4)	

Concern that smoking presence of your children will harm their health	Unconcerned	48	9.3	(5.3–15.7)	326	29.2	(23.5–35.7)	374	21.8	(16.7–28.0)	<0.001
	Moderate	121	24.4	(18.1–32.0)	557	39.3	(34.5–44.4)	678	33.8	(29.9–37.9)	
	Very/extremely	337	66.3	(58.7–73.2)	436	31.4	(26.4–37.0)	773	44.4	(38.7–50.3)	

Ban on smoking in home	No rules	150	27.2	(21.3–34.0)	684	50.7	(45.7–55.7)	834	42.1	(37.1–47.4)	<0.001
	Partial ban	42	10.3	(6.9–15.0)	179	13.9	(10.3–18.4)	221	12.6	(10.1–15.5)	
	Complete ban	321	62.5	(55.3–69.2)	526	35.4	(30.6–40.6)	847	45.3	(40.8–49.9)	

**Table 3.** Support for smoking bans in different venues and knowledge and attitudes about tobacco smoke pollution (weighted estimates), by highest level of education.

Measure		Illiterate			1 to 8 years			9 years or more			Rao-Scott χ^2^ Test
		Freq.	%	(95% CI)	Freq.	%	(95% CI)	Freq.	%	(95% CI)	p-value
Restrictions on smoking in hospitals	Complete ban	623	99.6	(98.0–100.0)	969	98.8	(97.2–99.6)	301	95.6	(90.7–98.3)	0.003
	Otherwise	3	0.4	(0.0–2.0)	11	1.2	(0.4–2.8)	11	4.4	(1.7–9.3)	

Restrictions on smoking in workplaces	Complete ban	501	82.2	(75.2–87.6)	848	87.8	(82.5–91.7)	280	90.4	(85.3–93.8)	0.031
	Otherwise	108	17.8	(12.4–24.8)	130	12.2	(8.3–17.5)	29	9.6	(6.2–14.7)	

Restrictions on smoking in restaurants	Complete ban	427	68.9	(60.4–76.2)	743	78.4	(73.4–82.7)	249	78.2	(70.7–84.2)	0.039
	Otherwise	182	31.1	(23.8–39.6)	232	21.6	(17.3–26.6)	61	21.8	(15.8–29.3)	

Restrictions on smoking in public transport	Complete ban	621	99.3	(97.7–99.9)	975	99.5	(98.9–99.9)	307	98.6	(95.7–99.8)	0.327
	Otherwise	4	0.7	(0.1–2.3)	8	0.5	(0.1–1.1)	5	1.4	(0.2–4.3)	

Restrictions on smoking in schools	Complete ban	609	97.4	(94.9–98.9)	932	94.8	(91.5–96.9)	291	93.7	(88.7–96.6)	0.031
	Otherwise	18	2.6	(1.1–5.1)	52	5.2	(3.1–8.5)	20	6.3	(3.4–11.3)	

Smoked tobacco is addictive	Agree/Strongly Agree	605	96.6	(94.1–98.2)	932	95.2	(92.2–97.3)	297	97.5	(95.1–98.9)	0.243
	Otherwise	22	3.4	(1.8–5.9)	41	4.8	(2.7–7.8)	13	2.5	(1.1–4.9)	

Second-hand smoke causes lung cancer in non-smokers	Yes	531	83.4	(75.0–89.4)	862	89.4	(85.6–92.2)	294	95.2	(91.4–97.6)	<0.001
	No	42	7.5	(3.9–13.8)	49	4.4	(2.3–7.5)	12	4.2	(1.8–8.3)	
	Don’t know	60	9.1	(6.0–13.6)	58	6.3	(4.5–8.8)	3	0.6	(0.1–2.3)	

Second-hand smoke causes asthma in children	Yes	540	84.7	(77.1–90.2)	891	93.3	(89.8–95.6)	299	96.2	(92.9–98.2)	
	No	29	6.0	(2.9–12.2)	32	2.5	(1.2–4.4)	6	1.8	(0.5–4.4)	<0.001
	Don’t know	63	9.3	(5.9–14.4)	45	4.3	(2.3–7.2)	5	2.1	(0.5–5.7)	

Concern that smoking in the presence of your children will harm their health	Unconcerned	173	36.2	(29.2–43.7)	158	16.6	(11.3–23.8)	43	14.8	(9.3–22.9)	<0.001
	Moderate	225	35.6	(29.7–41.9)	386	37.9	(32.1–44.2)	67	19.3	(13.1–27.4)	
	Very/extremely	179	28.3	(21.0–36.8)	405	45.5	(40.2–50.8)	187	65.9	(55.5–74.9)	

Ban on smoking in home	No rules	311	49.3	(43.4–55.3)	441	45.4	(39.5–51.4)	81	20.8	(14.0–29.9)	<0.001
	Partial ban	83	13.9	(10.3–18.7)	94	10.9	(6.9–16.9)	44	15.0	(10.5–21.1)	
	Complete ban	230	36.7	(32.0–41.8)	436	43.7	(37.8–49.8)	180	64.1	(54.7–72.6)	

**Table 4.** Household smoking conditions of Bangladeshi homes with children by highest level of education (weighted).

Measure		Illiterate			1 to 8 years			9 years or more			Rao-Scott χ^2^ Test
		Freq.	%	(95% CI)	Freq.	%	(95% CI)	Freq.	%	(95% CI)	p-value
Number of friends who smoke	0 or 1	33	7.1	(3.5–14.0)	28	4.7	(2.7–7.5)	12	4.3	(1.3–10.0)	0.401
	2 to 5	492	92.9	(86.0–96.5)	809	95.3	(92.5–97.3)	240	95.7	(90.0–98.7)	

Living with other adult smokers	No/no other smokers in home	333	55.9	(44.1–67.0)	526	63.0	(56.9–68.7)	170	65.1	(58.0–71.6)	0.205
	At least 1 other smoker in home	308	44.1	(33.0–55.9)	466	37.0	(31.3–43.1)	142	34.9	(28.4–42.0)	

Total smokers in the home	0	43	12.5	(7.1–21.0)	129	29.0	(21.7–37.5)	60	38.2	(27.8–49.8)	<0.001
	1	357	52.8	(37.8–67.3)	470	38.8	(28.5–50.3)	144	36.3	(26.1–48.0)	
	2 or more	241	34.7	(25.6–45.1)	393	32.2	(26.6–38.3)	108	25.5	(20.4–31.4)	

Concern about smoking in presence of children	Unconcerned/only a little concerned	173	36.2	(29.2–43.7)	158	16.6	(11.3–23.8)	43	14.8	(9.3–22.9)	<0.001
	Moderately concerned	225	35.6	(29.7–41.9)	386	37.9	(32.1–44.2)	67	19.3	(13.1–27.4)	
	Very/Extremely concerned	179	28.3	(21.0–36.8)	405	45.5	(40.2–50.8)	187	65.9	(55.5–74.9)	

Smoking rules in the home	No rules	311	49.3	(43.4–55.3)	441	45.4	(39.5–51.4)	81	20.8	(14.0–29.9)	<0.001
	Partial ban	83	13.9	(10.3–18.7)	94	10.9	(6.9–16.9)	44	15.0	(10.5–21.1)	
	Complete ban	230	36.7	(32.0–41.8)	436	43.7	(37.8–49.8)	180	64.1	(54.7–72.6)	

**Table 5.** The relationship between household smoking restrictions and the perception of risk for children’s exposure to TSP.

Measure		Unconcerned			Moderately Concerned			Very/Extremely Concerned			Rao-Scott χ^2^ Test
		Freq.	%	(95% CI)	Freq.	%	(95% CI)	Freq.	%	(95% CI)	p-value
Ban on smoking in home	No rules	204	56.2	(37.4–73.3)	360	50.7	(42.6–58.8)	208	27.4	(22.0–33.6)	< 0.001
	Partial ban	65	17.7	(10.3–28.6)	75	11.8	(7.7–17.7)	65	11.1	(7.0–17.2)	
	Complete ban	89	26.2	(16.6–38.8)	231	37.5	(30.6–45.0)	484	61.5	(55.9–66.8)	

**Table 6.** Predictors of concern (very or extremely) that smoking in the presence of children will harm their health: weighted logistic regression model.

	**Parameter**	**OR**	**(95% CI)**	**p-value**
Sex				
	Men		Reference	
	Women	0.97	(0.51–1.87)	0.935

Age				
	15 to 24		Reference	
	25 to 39	0.96	(0.50–1.86)	0.905
	40 to 54	0.86	(0.39–1.93)	0.719
	55+	0.97	(0.46–2.03)	0.939

Residence				
	Rural		Reference	
	Slums	1.57	(1.04–2.37)	0.032
	Urban, non-slum	1.37	(0.86–2.18)	0.191

Marital status				
	Single or divorced		Reference	
	Married	1.19	(0.63–2.27)	0.598

Education				
	Illiterate		Reference	
	1 to 8 years	1.94	(1.34–2.80)	<0.001
	9 years or more	4.07	(2.21–7.52)	<0.001

Smoking status				
	Non-smoker		Reference	
	Smoker	0.24	(0.14–0.41)	<0.001

Number of children in home				

	One		Reference	
	Two	0.97	(0.68–1.37)	0.845
	Three or more	0.89	(0.60–1.32)	0.567

Age of youngest child				
	5 or younger		Reference	
	6 to 13	1.41	(0.98–2.03)	0.068

Living with other adult smokers				
	None/no one		Reference	
	At least one (other) smoker	1.14	(0.83–1.54)	0.421

**Table 7.** Predictors of complete home smoking bans: weighted logistic regression model.

**Parameter**		**OR**	**(95% CI)**	**p-value**
Sex				
	Men		Reference	
	Women	0.94	(0.52–1.71)	0.846

Age				
	15 to 24		Reference	
	25 to 39	1.34	(0.64–2.84)	0.439
	40 to 54	1.45	(0.69–3.07)	0.332
	55+	1.21	(0.56–2.62)	0.622

Residence				
	Rural		Reference	
	Slums	1.78	(1.11–2.87)	0.018
	Urban, non-slum	1.64	(1.00–2.69)	0.050

Marital status				
	Single or divorced		Reference	
	Married	0.93	(0.61–1.43)	0.751

Education				
	Illiterate		Reference	
	1 to 8 years	1.00	(0.74–1.35)	0.988
	9 years or more	1.94	(1.26–3.00)	0.003

Smoking status				
	Non-smoker		Reference	
	Smoker	0.40	(0.24–0.66)	< 0.001

Number of children in home				
	One		Reference	
	Two	1.01	(0.69–1.48)	0.961
	Three or more	0.95	(0.62–1.45)	0.814

Age of youngest child				
	5 or younger		Reference	
	6 to 13	1.01	(0.72–1.40)	0.966

Living with other adult smokers				
	None/no others		Reference	
	At least one (other) smoker	0.79	(0.54–1.16)	0.228

Concern that smoking in presence of children will harm health				
	Unconcerned		Reference	
	Moderately concerned	1.73	(0.85–3.52)	0.131
	Very/extremely concerned	3.25	(1.60–6.58)	0.001
